# Unit Operation Optimization for the Manufacturing of Botanical Injections Using a Design Space Approach: A Case Study of Water Precipitation

**DOI:** 10.1371/journal.pone.0104493

**Published:** 2014-08-07

**Authors:** Xingchu Gong, Huali Chen, Teng Chen, Haibin Qu

**Affiliations:** Pharmaceutical Informatics Institute, College of Pharmaceutical Sciences, Zhejiang University, Hangzhou, China; University of Nebraska Medical Center, United States of America

## Abstract

Quality by design (QbD) concept is a paradigm for the improvement of botanical injection quality control. In this work, water precipitation process for the manufacturing of Xueshuantong injection, a botanical injection made from *Notoginseng Radix et Rhizoma*, was optimized using a design space approach as a sample. Saponin recovery and total saponin purity (TSP) in supernatant were identified as the critical quality attributes (CQAs) of water precipitation using a risk assessment for all the processes of Xueshuantong injection. An Ishikawa diagram and experiments of fractional factorial design were applied to determine critical process parameters (CPPs). Dry matter content of concentrated extract (DMCC), amount of water added (AWA), and stirring speed (SS) were identified as CPPs. Box-Behnken designed experiments were carried out to develop models between CPPs and process CQAs. Determination coefficients were higher than 0.86 for all the models. High TSP in supernatant can be obtained when DMCC is low and SS is high. Saponin recoveries decreased as DMCC increased. Incomplete collection of supernatant was the main reason for the loss of saponins. Design space was calculated using a Monte-Carlo simulation method with acceptable probability of 0.90. Recommended normal operation region are located in DMCC of 0.38–0.41 g/g, AWA of 3.7–4.9 g/g, and SS of 280–350 rpm, with a probability more than 0.919 to attain CQA criteria. Verification experiment results showed that operating DMCC, SS, and AWA within design space can attain CQA criteria with high probability.

## Introduction

Quality by Design (QbD) is a systematic approach based on knowledge management and risk management [1]–[3]. It has become a paradigm for pharmaceutical industry to establish analytical methods [4]–[6], develop new manufacturing processes [7]–[12], and optimize existing manufacturing processes [13]. Manufacturing process optimization under the framework of QbD will benefit the target patient population, the pharmaceutical industry, and the regulation agency. Implementation of QbD concept usually contains several steps, such as critical quality attribute (CQA) definition, risk assessment, critical process parameter (CPP) determination, design space development, control strategy design, and continual improvement in drug lifecycle [13]–[15].

To keep batch-to-batch consistency, design space development is very important in the implementation of QbD concept. Parameter variations within design space are not considered to affect product quality. Quantitative models between process parameters and process CQAs should be established to develop design space. CPPs can be determined using risk assessment or design of experiments. Plackett–Burman design or fractional factorial design are usually applied to select CPPs [16]. In order to obtain design space, response surface methodology, such as central composite design [17] or Box-Behnken design [18], is widely applied. Recently, Rozet et al. suggested that design space is “a multivariate domain of input factors ensuring that critically chosen responses are included within predefined limits with an acceptable level of probability” [16]. The Monte-Carlo method is adopted by several researchers to calculate the probability in the development of analytical methods [19], [20].

Botanical drugs have gained increasing popularity in recent years. The compositions of most botanical drugs are very complicated. There is usually more than one distinct active ingredient in a botanical drug. Accordingly, the quality control of botanical drugs is difficult. Recently, Zhang et al. applied the QbD concept to develop botanical drug manufacturing processes [15].

**Figure 1:**
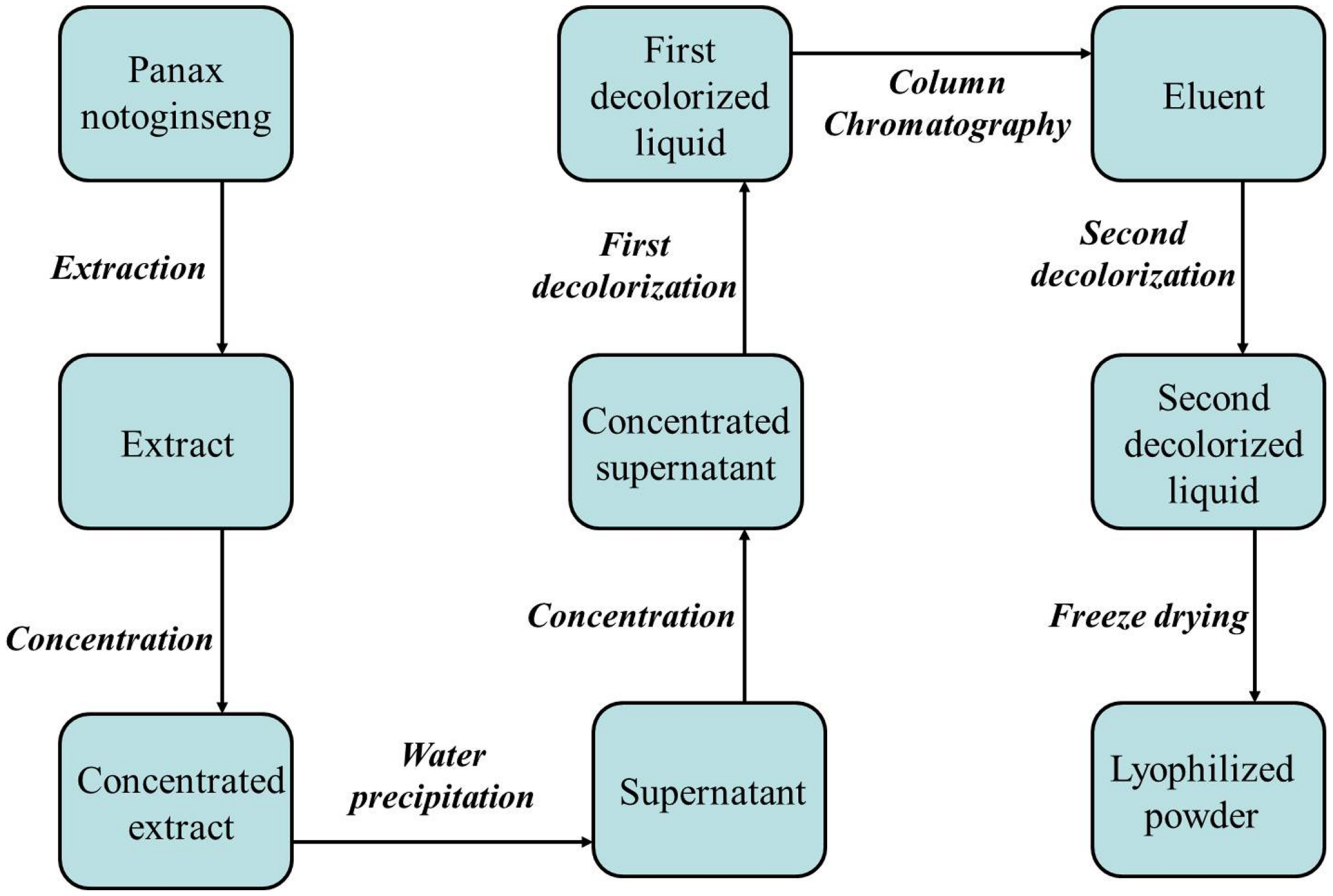
The manufacturing processes of Xueshuantong powder.

In this work, water precipitation process in the manufacturing of Xueshuantong powder was optimized in the framework of QbD concept as a sample. Xueshuantong powder was applied to treat retinal vein occlusion and cerebrovascular disease in China [21]. It is made from *Notoginseng Radix et Rhizoma* (Sanqi) through a series of unit operations, including extraction with a mixed ethanol-water solution, concentration, water precipitation, decolorization, column chromatography, and freeze drying, as shown in Figure 1 [22]. The active pharmaceutical ingredients (APIs) of Xueshuantong powder are saponins, including Notoginsenoside R_1_, ginsenoside Rg_1_, ginsenoside Rb_1_, ginsenoside Rd, and ginsenoside Re.

The mixed solvent of ethanol and water is widely applied to extract active ingredients from medicinal herbs [23]–[26]. Water precipitation is a precipitation process usually used in the treatment of plant extracts extracted using ethanol solution. It can be realized by simply adding water into the concentrated extracts of plants. The purpose of this process is to remove impurities with weak polarity. In the production of Xueshuantong injection, the loss of saponins are observed in water precipitation. Therefore water precipitation process affects both drug efficacy and drug safety. However, there are very few publications on water precipitation.

In this work, water precipitation was optimized using a design space approach. Process CQAs of water precipitation were obtained through risk assessment. Process parameters are also discussed in risk assessment. Fractional factorial designed experiments were used to determine CPPs of water precipitation. Design space was calculated based on the Box-Behnken designed experimental results with a Monte-Carlo simulation method. Finally, design space was verified.

## Experimental

### Materials and Chemicals

Sanqi was collected from Wenshan of Yunnan Province (China) and purchased from Zhejiang Chinese Medical University Medical Pieces Co. Ltd. (Hangzhou, China). No specific permissions were required for the described field studies. The locations are neither privately owned nor protected by the Chinese government. No endangered or protected species were sampled.

Standard substances of notoginsenoside R_1_, ginsenoside Rg_1_, ginsenoside Re, ginsenoside Rb_1_, and ginsenoside Rd were purchased from Shanghai Winherb Pharmaceutical Technology Development. Co., Ltd. (Shanghai, China). Acetonitrile (HPLC grade) and methanol (HPLC grade) were obtained from Merck (Darmstadt, Germany). The formic acid (HPLC grade) was purchased from Tedia (Darmstadt, Germany). Deionized water was produced using a Milli-Q academic water purification system (Milford, MA, USA).

### Procedures

Sanqi was crushed and sieved. After that, a mixed solvent of 60% ethanol and 40% water (v/v) was used to extract Sanqi with water bath (W501, Shanghai Shen Sheng Biotechnology Co., Ltd) operated at 95°C for three times. The obtained extracts were mixed and concentrated. The dry matter content of concentrated extract (DMCC) was 509.1 mg/g. Concentrated extracts with lower DMCC were obtained by dilution with water.

Water was added to concentrated extract in a conical flask under stirring with a roller pump (BT300-2J, Baoding Longer Precision Pump Co., Ltd.) at a desired flowrate. After adding water, the stirring was kept for 10 min. The flask then was refrigerated in a low-temperature thermostat bath (THD-1008W, Ningbo Tianheng Instrument Factory) for the desired time at the desired temperature. Finally, the supernatant was collected and weighed. Saponin contents and dry matter content of the supernatant then were determined.

### Experimental design

Six parameters were investigated, including DMCC, amount of water added (AWA), stirring speed (SS), refrigeration temperature, refrigeration time, and the flowrate of water addition. The coded and uncoded values of parameters are listed in Table 1. Fractional factorial designed experiments were applied to select CPPs, as seen in Table 2. Box-Behnken designed experiments were applied to obtain the quantitative models between CQAs and CPPs, as seen in Table 3. After the development of design space, verification experiments with conditions listed in Table 4 were carried out. Verification experiments were repeated three times.

**Table 1:** Coded and uncoded values for the factors.

Parameters	Symbols	Coded values	
		−1	1
DMCC (g/g)	A	0.34	0.55
AWA (g/g)	B	2.5	5.5
Stirring speed (rpm)	C	200	400
Refrigeration temperature (°C)	D	5.0	30.0
Refrigeration time (h)	E	12	48
Flowrate (ml/min)	F	3.0	7.0

**Table 2:** Fractional factorial designed experiments and results.

Run	A	B	C	D	E	F	Recovery of each saponin in supernatant (%)					Total saponin purity in supernatant (%)
							Notoginsenoside R_1_	Ginsenoside Rg_1_	Ginsenoside Re	Ginsenoside Rb_1_	Ginsenoside Rd	
1	0.55	5.5	400	5.0	12	3.0	92.6	91.3	92.8	94.3	96.0	39.2
2	0.34	2.5	400	5.0	48	3.0	89.1	88.5	85.6	93.1	98.6	39.8
3	0.34	5.5	400	30.0	48	3.0	94.0	93.6	90.4	97.7	103.2	42.1
4	0.55	5.5	200	30.0	12	7.0	90.5	91.3	90.7	94.6	100.9	38.5
5	0.34	5.5	400	5.0	12	7.0.	93.3	93.2	88.4	96.8	99.2	40.6
6	0.55	2.5	400	30.0	12	3.0	84.6	84.0	85.0	90.9	104.8	42.1
7	0.55	5.5	400	30.0	48	7.0	92.3	91.9	90.2	95.6	101.2	43.8
8	0.34	5.5	200	5.0	48	7.0	92.8	93.3	89.7	82.7	78.6	37.6
9	0.34	2.5	400	30.0	12	7.0	89.8	90.0	85.2	95.2	100.6	42.3
10	0.55	2.5	400	5.0	48	7.0	84.8	84.1	84.5	91.3	99.9	39.3
11	0.55	2.5	200	30.0	48	3.0	84.7	84.1	85.1	89.6	96.9	38.3
12	0.55	5.5	200	5.0	48	3.0	90.8	90.7	90.4	92.9	97.9	38.3
13	0.55	2.5	200	5.0	12	7.0	85.5	85.0	84.6	91.0	99.7	39.6
14	0.34	2.5	200	30.0	48	7.0	88.3	88.6	83.7	94.1	101.8	38.0
15	0.34	5.5	200	30.0	12	3.0	94.2	94.3	89.5	98.7	106.2	41.4
16	0.34	2.5	200	5.0	12	3.0	88.9	88.6	84.2	93.5	101.8	41.0

**Table 3:** Box-Behnken designed experiments and results.

Run	A	B	C	Recovery of each saponin in supernatant/(%)					Total saponin purity in supernatant (%)
				Notoginsenoside R_1_	Ginsenoside Rg_1_	Ginsenoside Re	Ginsenoside Rb_1_	Ginsenoside Rd	
1	0.34	4.0	400	88.8	88.4	90.0	90.5	97.7	43.2
2	0.34	5.5	300	91.5	91.6	94.1	94.4	99.6	41.8
3	0.34	2.5	300	86.7	87.2	89.9	90.5	95.8	41.5
4	0.42	2.5	200	86.2	85.3	88.0	89.5	90.7	40.8
5	0.42	2.5	400	87.6	85.7	86.1	88.7	92.2	43.1
6	0.34	4.0	200	89.9	90.3	89.0	93.8	94.3	38.9
7	0.52	4.0	200	87.5	86.8	90.6	91.2	93.2	41.1
8	0.52	2.5	300	80.8	78.7	81.2	81.9	83.7	40.2
9	0.52	5.5	300	87.0	85.4	87.3	88.0	90.6	39.8
10	0.42	4.0	300	89.4	88.9	89.7	91.9	88.9	41.5
11	0.42	5.5	400	89.7	89.4	91.4	92.0	93.9	41.1
12	0.42	5.5	200	92.1	91.6	92.9	93.4	96.4	41.2
13	0.52	4.0	400	84.9	82.8	84.2	87.0	88.8	39.4
14	0.42	4.0	300	88.2	88.1	89.7	90.9	90.9	41.9
15	0.42	4.0	300	88.2	87.5	88.8	88.9	88.0	40.2
16	0.42	4.0	300	90.2	88.4	88.3	91.3	91.9	41.3

**Table 4:** Conditions and results of verification experiments.

No.		V1	V2
Concentrated extract amount (g)		3.0	3.0
DMCC (g/g)		0.52	0.41
AWA (g/g)		2.5	3.7
SS (rpm)		400	320
Probability		0.00	0.95
Within design space		No	Yes
Notoginsenoside R_1_ recovery (%)	Experimental value	84.1±1.1	89.2±0.5
	Predicted value	82.3	88.6
Ginsenoside Rg_1_ recovery (%)	Experimental value	84.6±1.0	87.1±0.2
	Predicted value	79.3	87.7
Ginsenoside Re recovery (%)	Experimental value	83.9±1.9	87.9±0.6
	Predicted value	79.9	88.6
Ginsenoside Rb_1_ recovery (%)	Experimental value	89.1±1.1	91.7±0.2
	Predicted value	83.1	90.3
Ginsenoside Rd recovery (%)	Experimental value	90.5±1.4	92.3±0.5
	Predicted value	86.4	90.0
Total saponin purity in supernatant (%)	Experimental value	40.4±0.2	40.3±0.5
	Predicted value	40.3	41.5

### Analytical methods

The quantitative analysis of saponins was performed using a UPLC method. A Waters Acquity UPLC system (Waters, Milford, MA) coupled with a Waters Acquity CSH C18 column (50 mm×2.1 mm, 1.7 µm) was used. The mobile phase consisted of 0.01% (v/v) formic acid in water (FA) and 0.01% (v/v) formic acid in acetonitrile (FB). The separation was achieved using a gradient elution program as follow: 19–20% FB at 0–6 min, 20–31% FB at 6–8.5 min, 31–33% FB at 8.5–11 min, 33–90% FB at 11–17 min, and 90–90% FB at 17–19 min. The solvent flow rate was 0.35 ml/min, and the column temperature was kept at 45°C. The detection wavelength was 203 nm. Dry matter content was determined gravimetrically using a precision electronic balance (AB204-N, Mettler Toledo Shanghai Co., Ltd). Before weighed, samples were dried at 105°C in an oven (DZF-6050, Shanghai Jing Hong Laboratory Instrument Co., Ltd.) for 3 h and then kept in a desiccator for 0.5 h.

### Data processing

The recovery of saponins (SR) are defined as following equation.
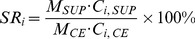
(1)where M and C refer to the mass and concentration, respectively; subscript SUP and CE are supernatant and concentrated extract, respectively; subscript i (i = 1 to 5) represents notoginsenoside R_1_, ginsenoside Rg_1_, ginsenoside Re, ginsenoside Rb_1_, and ginsenoside Rd, respectively. The content of total saponin (C_TS_) in supernatant or concentrated extract was calculated using Equation 2.
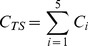
(2)The purity of total saponin (TSP) was calculated using Equation 3.
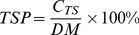
(3)where DM is the dry matter content of supernatant or concentrated extract.

Design Expert V8.0.6.1 (State-Ease Inc., MN) was used to analyze the results of fractional factorial design and Box-Behnken design. In the selection of CPPs, only the main effects of factors were considered, as shown in Equation 4.
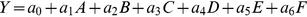
(4)where Y is the response; A, B, C, D, E, and F represent parameters; a_0_ is a constant; a_1_ to a_6_ are regression coefficients. The significance level was set to 0.05. Equation 5 was used to model the results of Box-Behnken experiments.
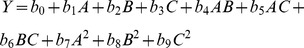
(5)where b_0_ is a constant, and b_1_ to b_9_ are regression coefficients. All the parameters were coded before modeling.

The design space was calculated using a Monte-Carlo method with a self-written program of Matlab (R2010b,Version 7.11, MathWorks, USA). Considering the sampling error, saponin contents and dry matter contents in supernatant were assumed to follow a normal distribution. To generate random data of saponin contents and dry matter contents, the average values and standard deviations were required. The experimental values of saponin contents and dry matter contents were used as the average values in simulation. The relative standard deviations (RSD) of saponin contents and dry matter contents of supernatants were considered to be the same with the RSD values of the center point in experimental design. Then the standard deviations can be calculated according to average values and RSD values. Coded values were used in the calculation. Therefore the ranges of DMCC, AWA, and SS were within −1 and 1. The calculation step sizes for DMCC, AWA, and SS were 0.02, 0.02, and 0.02, respectively. Saponin recovery and TSP in supernatant were calculated in each simulation. Simulation was repeated 50000 times to calculate reliable probability values. The acceptable level of probability for design space was set as 0.90.

Supernatant remaining in the pores or surface of precipitation (SRP) was not collected. The mass of SRP (M_SRP_) was calculated using Equation 6.
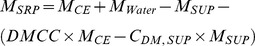
(6)The ratio of saponins in SRP (SRSRP) was calculated using Equation 7.
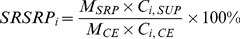
(7)


## Results and Discussion

### Process CQA determination

There are many CQAs for Xueshuantong powder, including color, water content, pH value, saponin purity in dry powder, fingerprint similarity, abnormal toxicity, residue on ignition, insoluble particles, bacterial content, heavy metals, and harmful elements. Heavy metals and harmful elements are generally considered to be affected by raw material quality. Bacterial content was controlled by producing Xueshuantong powder in sterile conditions. Process influences on other drug CQAs are identified using risk assessment based on experiences, as seen in Table 5. As one of the unit operations in the manufacturing of Xueshuantong powder, water precipitation is considered to affect saponin purity in dry powder and fingerprint similarity. Higher active ingredient recovery and higher TSP in supernatant both help to realize higher saponin purity in dry powder. Higher TSP in supernatant also means higher removal of impurities, which affects fingerprint similarity. Therefore saponin recovery and TSP in supernatant were identified as the process CQAs in this work. Their criteria are listed in Table 6.

**Table 5:** Risk assessment to identify process influences.

Drug quality attributes	Extraction	Concentration	Water precipitation	First decolorization	Column chromatography	Second decolorization	Freeze drying
Color	+	−	−	++	−	++	−
Water content	−	−	−	−	−	−	++
pH value	−	−	−	−	+	−	−
Saponin content in dry powder	+	−	+	+	++	+	−
Fingerprint similarity	+	−	+	+	++	+	−
Abnormal toxicity	−	−	−	−	+	−	−
Residue on ignition	−	−	−	−	+	−	−
Insoluble particles	−	−	−	−	+	−	−

++, +, and − refer to high influence, moderate influence, and low influence, respectively.

**Table 6:** Criteria of process CQAs.

CQAs	Lower control limit (%)	Upper control limit (%)
Notoginsenoside R_1_ recovery	85	92
Ginsenoside Rg_1_ recovery	85	91
Ginsenoside Re recovery	85	94
Ginsenoside Rb_1_ recovery	85	95
Ginsenoside Rd recovery	85	99
TSP in supernatant	40	43

### Selection of CPPs

**Figure 2:**
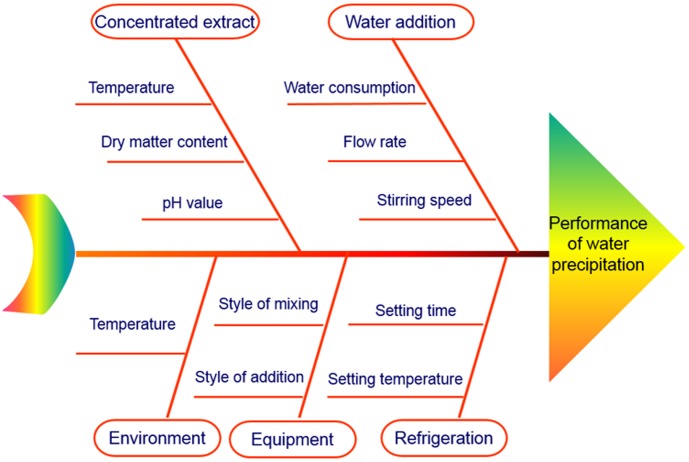
Ishikawa diagram of water precipitation process.

An Ishikawa diagram analysis was performed to find out process parameters that affect the CQAs of water precipitation, as seen in Figure 2. The risk severity of four parameters of concentrated extract temperature, concentrated extract pH value, environmental temperature, and water addition style is low. Parameter fail probability of water addition style, and style of mixing is very low. The detectability for concentrated extract temperature, and environmental temperature is high. Therefore, the risk caused by these five parameters are low.

**Figure 3:**
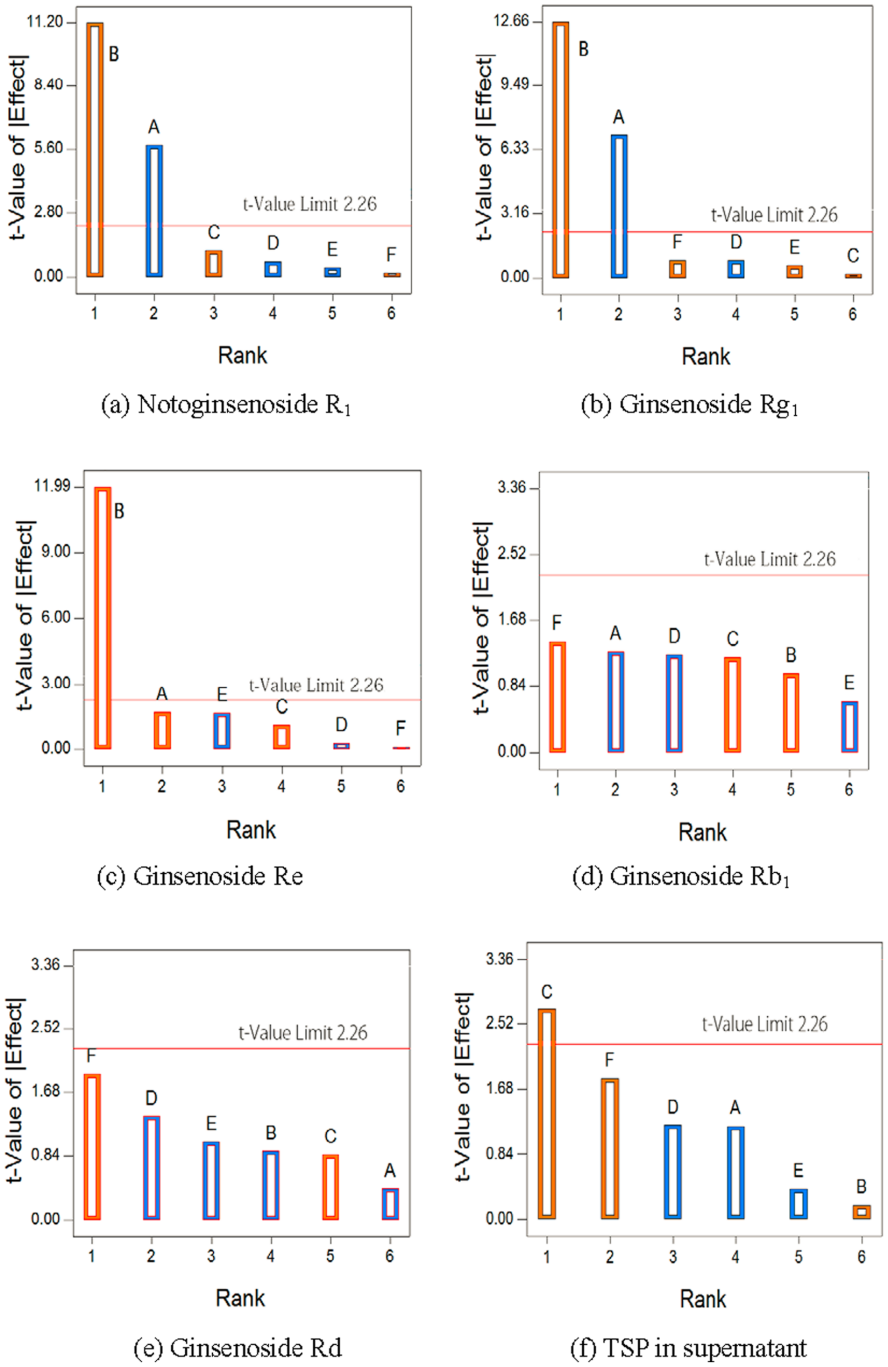
Pareto chart of parameters.

The other six parameters of AWA, SS, DMCC, refrigeration temperature, refrigeration time, and water addition flow rate were investigated using fractional factorial design. The results of fractional factorial design experiments are listed in Table 2. The Pareto chart for saponin recovery and TSP in supernatant are shown in Figure 3. In Figure 3(a) and 3(b), DMCC and AWA remarkably affected the recoveries of notoginsenoside R_1_ and ginsenoside Rg_1_. Ginsenoside Re recovery was mainly affected by AWA, as seen in Figure 3(c). In Figure 3(f), SS significantly affected TSP in supernatant. The other three factors, including refrigeration temperature, refrigeration time, and the flow rate of water were insignificant on process CQAs. Therefore DMCC, AWA, and SS were selected as CPPs.

### The effects of CPPs on process CQAs

The experimental results of Box-Behnken design are displayed in Table 3. The recoveries of all the five saponins are higher than 78%, which means most active ingredients can be recovered in water precipitation. TSP in supernatant varied from 38.8% to 43.2%. Because TSP in concentrated extract was 36.7%, it can be concluded that TSP increases after water precipitation. Response surface models were developed to obtain quantitative relationships between CQAs and CPPs. The estimated regression coefficients are listed in Table 7. Analysis of variance (ANOVA) was performed, and p values of parameters are also listed in Table 7. For all the models, determination coefficients (R^2^) are larger than 0.86, which means most variations of process CQAs can be explained by DMCC, AWA, and SS. Models are significant with p-values less than 0.05. In Table 7, DMCC are significant for all the CQAs. AWA are significant for all the recoveries of saponins. The quadratic term of DMCC shows significant influences on the recovery of notoginsenoside R_1_ and ginsenoside Rg_1_. The purity of total saponin in supernatant is mainly affected by DMCC and the interaction term of DMCC and SS.

**Table 7:** Estimated parameter values and ANOVA results.

Model terms	Recovery										TSP in supernatant	
	Notoginsenoside R_1_		Ginsenoside Rg_1_		Ginsenoside Re		Ginsenoside Rb_1_		Ginsenoside Rd		Estimate	Prob>|t|
	Estimate	Prob>|t|	Estimate	Prob>|t|	Estimate	Prob>|t|	Estimate	Prob>|t|	Estimate	Prob>|t|		
Constant	88.79	--------	87.92	--------	88.87	--------	90.47	--------	89.46	--------	41.17	--------
A	−2.08	0.0016*	−2.98	0.0002*	−2.48	0.0063*	−2.63	0.0059*	−3.91	0.0018*	−0.60	0.0353*
B	2.40	0.0008*	2.68	0.0004*	2.61	0.0051*	2.19	0.0135*	2.32	0.0197*	−0.23	0.3484
C	−0.60	0.1667	−0.99	0.0399*	−1.20	0.0948	−1.23	0.1007	−0.35	0.6500	0.51	0.0624
A×B	0.38	0.5076	0.58	0.3166	0.47	0.5974	0.57	0.5432	0.82	0.4573	−0.18	0.5887
A×C	−0.42	0.4675	−0.59	0.3070	−1.88	0.0697	−0.33	0.7258	−1.96	0.1070	−1.52	0.0028*
B×C	−0.93	0.1343	−0.63	0.2793	0.11	0.9058	−0.17	0.8511	−1.00	0.3734	−0.58	0.1125
A^2^	−1.60	0.0269*	−1.42	0.0397*	−0.82	0.3824	−0.88	0.3702	1.81	0.1365	−0.58	0.1195
B^2^	−0.70	0.2439	−0.79	0.1894	−0.075	0.9326	−0.87	0.3645	1.15	0.3096	0.24	0.4675
C^2^	0.58	0.3233	0.58	0.3207	0.39	0.6601	1.03	0.2939	2.22	0.0758	0.059	0.8571
Model P value	0.0057*		0.0018*		0.0310*		0.0487*		0.0235*		0.0277*	
*_R_* ^2^	0.9367		0.9577		0.8833		0.8616		0.8947		0.8881	

* p value less than 0.05.

**Figure 4:**
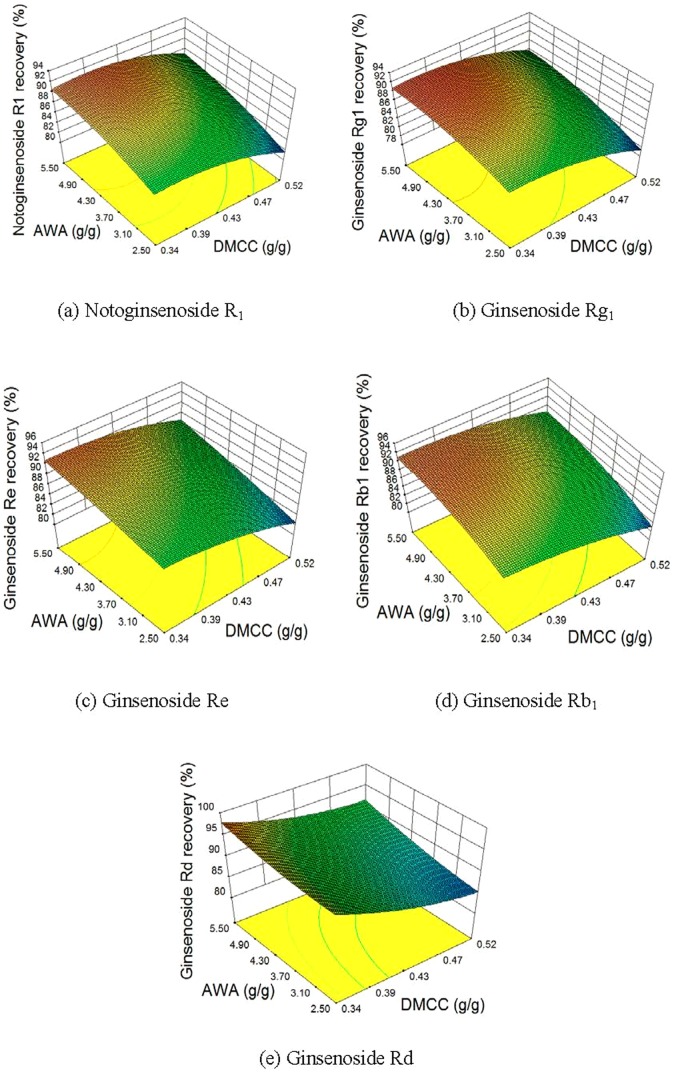
Effects of DMCC and AWA on saponin recoveries.

**Figure 5:**
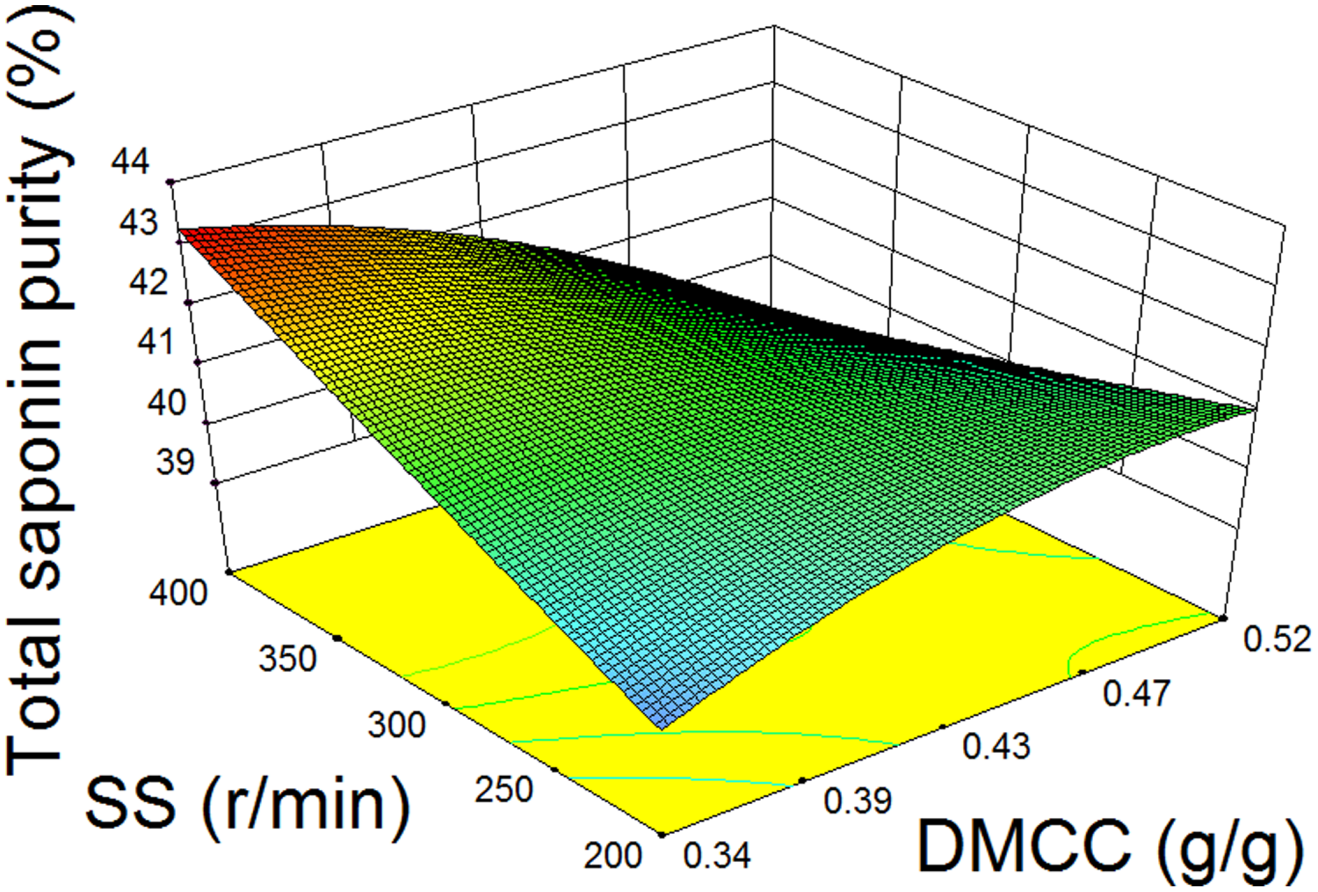
Effects of DMCC, and SS on TSP in supernatant.

Figure 4 shows the effects of DMCC and AWA on the recoveries of saponins. The increase of AWA can reduce saponin loss. However, saponin recoveries decrease as DMCC increases. The effects of CPPs on TSP in supernatant are shown in Figure 5. High TSP in supernatant can be obtained when DMCC is low and SS is high.

### Loss mechanism of saponins

**Figure 6:**
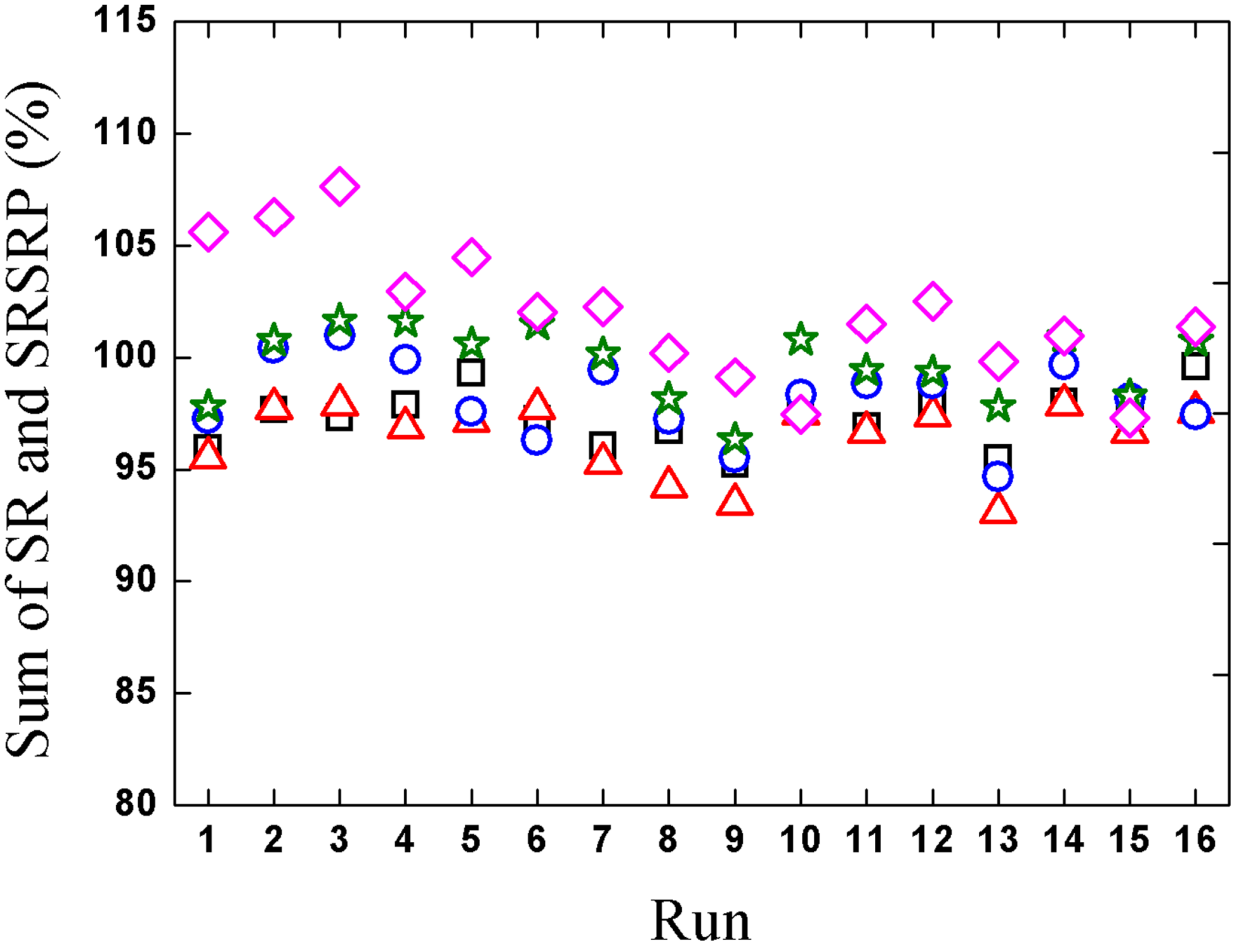
Sum of SR and SRSRP for different saponins.

Saponins will degrade under acidic, basic, or thermal conditions [27]–[29]. The solubilities of some saponins were also reported [30], [31]. It can be concluded that saponin solubility in water is not large. Theoretically, the loss of saponins in water precipitation may be caused by chemical reactions, precipitation, or the incomplete collection of supernatant. The sums of SR and SRSRP for each saponin are plotted in Figure 6. Most values of the sums of SR and SRSRP are within 100%±5%. It indicates that the incomplete collection of supernatant is the main reason for the loss of saponins. The uncollected supernatant mainly remained in the pores and surface of precipitation.

### Design space development and verification

**Figure 7:**
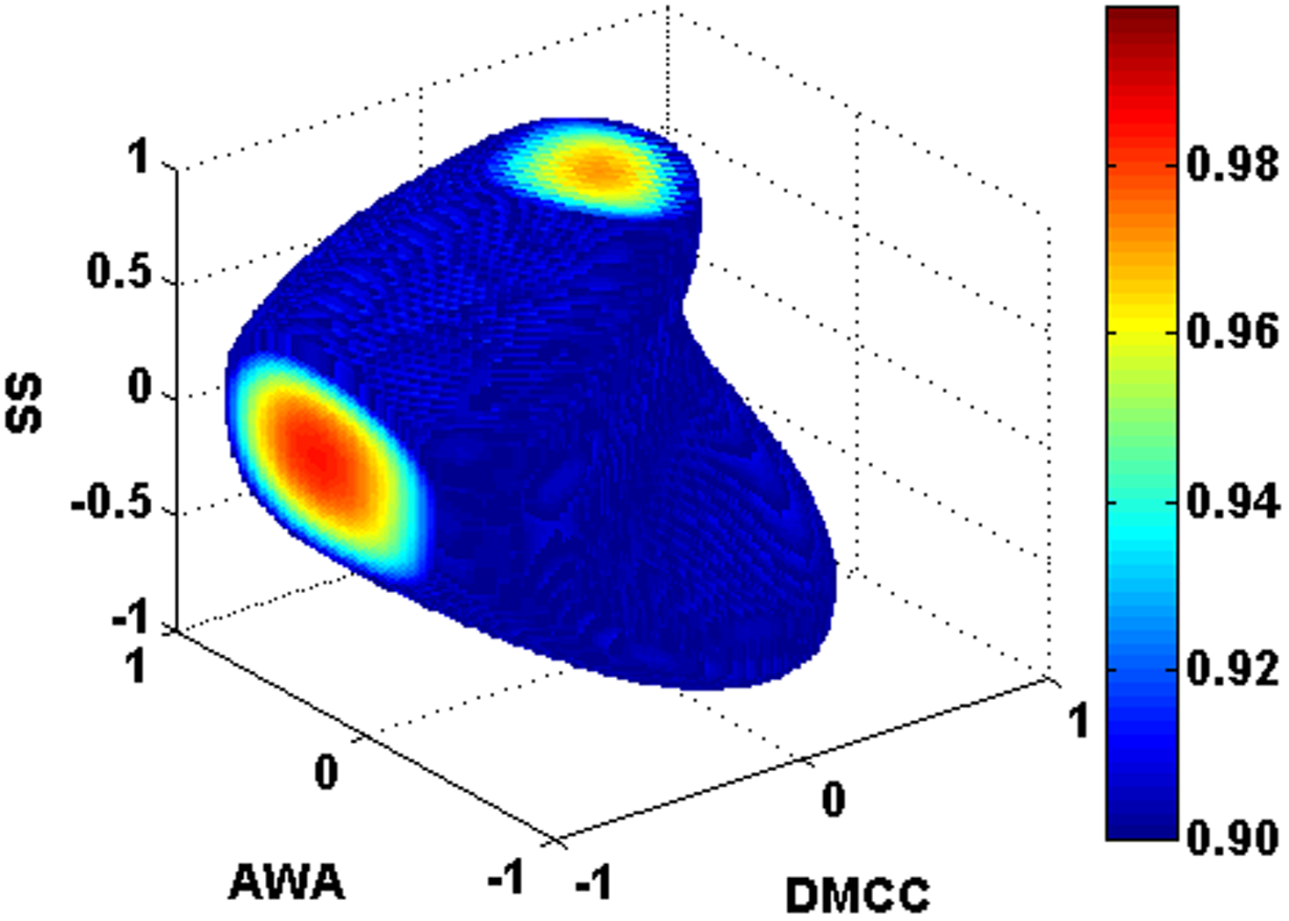
Design space obtained using Monte-Carlo simulations.

The calculated design space are shown in Figure 7. It can be concluded that the design space is an irregular polygon. Recommended normal operation region to attain CQA criteria are located in DMCC of 0.38–0.41 g/g, AWA of 3.7–4.9 g/g, and SS of 280–350 rpm with a probability more than 0.919. Verification experiments were carried out. The prediction results and experimental results of process CQAs are listed in Table 4. The recoveries of notoginsenoside R_1_, ginsenoside Rg_1_, and ginsenoside Re of Experiment V1 are lower than their criteria. While all the experimental values of Experiment V2 were in the ranges listed in Table 6. These results indicate that criteria of CQAs can be attained by operating CPPs within design space.

## Conclusions

In this work, water precipitation process for the manufacturing of Xueshuantong injection was optimized using a design space approach as a sample. Saponin recovery and the total saponin purity in supernatant were identified as the process CQAs of water precipitation using risk assessment. An Ishikawa diagram was applied to find out potential CPPs. DMCC, SS, and AWA were identified as CPPs using a fractional factorial design. Models between CPPs and process CQAs were developed with experimental results of Box-Behnken design. Determination coefficients were higher than 0.86 for all the models. High TSP in supernatant can be obtained when DMCC is low and SS is high. Saponin recoveries decrease as DMCC increases. The loss of saponins was mainly caused by the incomplete collection of supernatant. Design space was calculated with a Monte-Carlo simulation method. In the simulation, 0.90 was adopted as the acceptable probability for the attainment of CQA criteria. Recommended normal operation region are located in DMCC of 0.38–0.41 g/g, AWA of 3.7–4.9 g/g, and SS of 280–350 rpm, with a probability more than 0.919 to attain CQA criteria. Design space was verified and verification experimental results showed that operating DMCC, SS, and AWA within design space helps to attain process CQA criteria with high probability. The design space applied in this work can also be used to optimize other unit operations in pharmaceutical engineering.
